# Supplementary material for: Sex differences in genetic and environmental influences on frailty and its relation to body mass index and education
Source: Aging (Albany NY). 2021 Jul 6;13(13):16990–7023. doi: 10.18632/aging.203262 (PMC8312411; doi:10.18632/aging.203262)
Supplement: Supplementary Figures [file aging-13-203262-s001.pdf]

SUPPLEMENTARY FIGURES

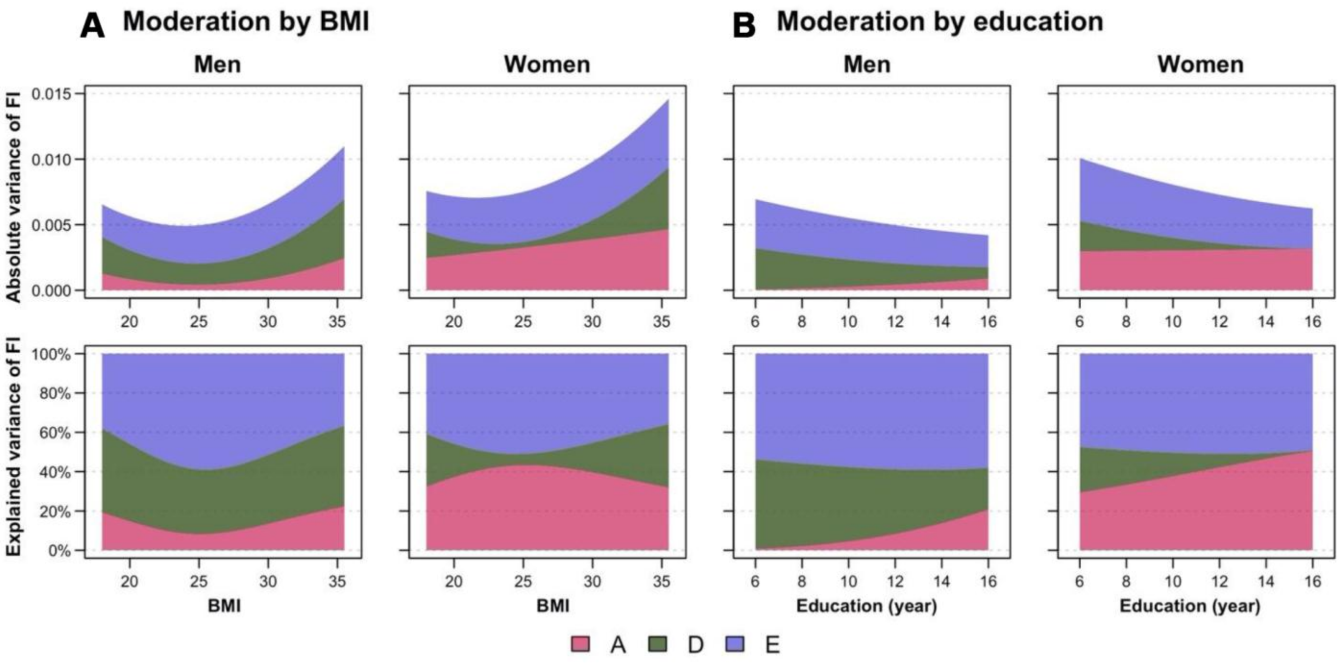

Supplementary Figure 1. Variance components of frailty index (FI) by (**A**) body mass index (BMI) and (**B**) education from moderation analysis, stratified by sex. First row shows the absolute variance of FI, while the second row shows the proportion of FI variance explained by additive genetic (A), dominance genetic (D) and unique environmental (E) factors, with changes in BMI and education. Variance estimates of moderation by BMI were obtained from the full ADE bivariate moderation model between FI and BMI; while the variance estimates of moderation by education were obtained from the ADE extended univariate moderation model between FI and education. Quantitative sex-differences were allowed in the models to obtain estimates separately for men and women. Models were adjusted for age.

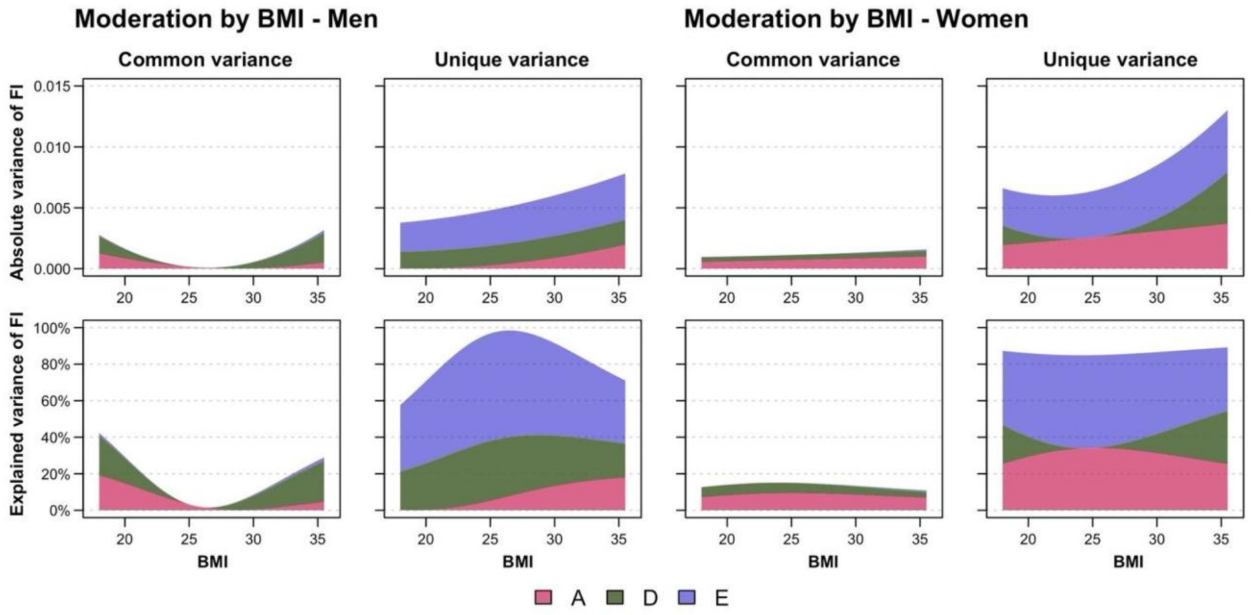

Supplementary Figure 2. Common and unique variance components of frailty index (FI) by body mass index (BMI) from moderation analysis, stratified by sex. First row shows the absolute variance of FI, while the second row shows the proportion of FI variance explained by additive genetic (A), dominance genetic (D) and unique environmental (E) factors, with changes in BMI levels. Variance estimates were obtained from the full ADE bivariate moderation model between FI and BMI. Quantitative sex-differences were allowed in the models to obtain estimates separately for men and women. Models were adjusted for age.

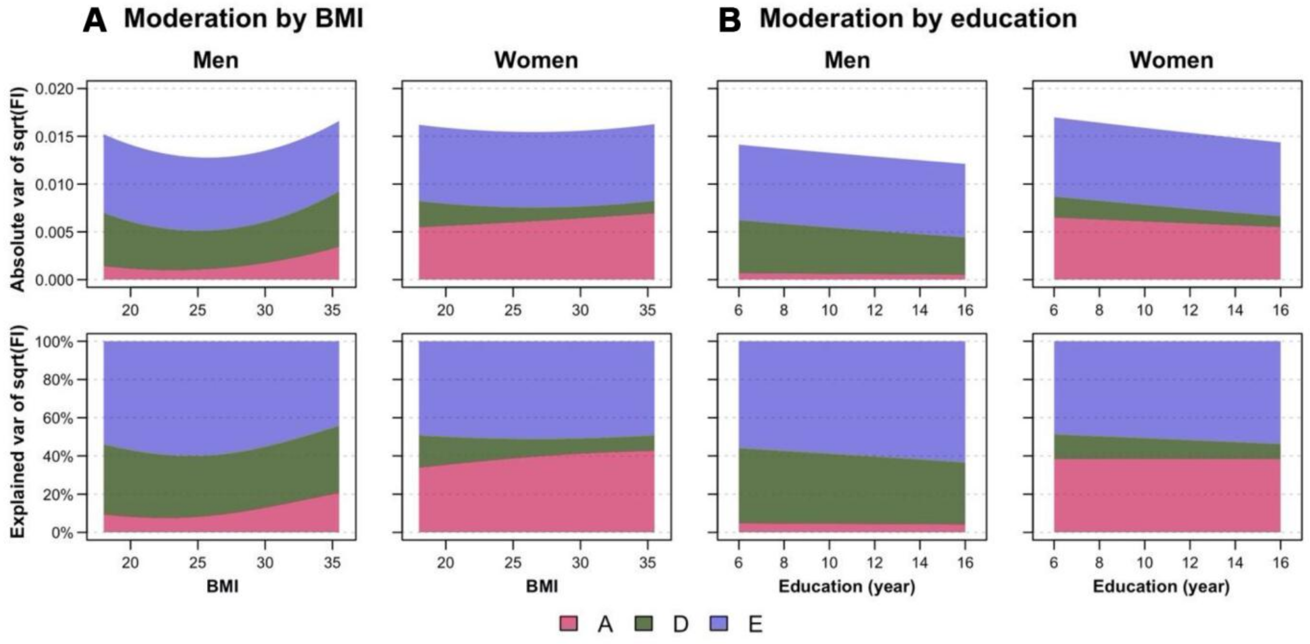

Supplementary Figure 3. Variance components of square-root transformed frailty index [sqrt(FI)] by (**A**) body mass index (BMI) and (**B**) education from moderation analysis, stratified by sex. First row shows the absolute variance of sqrt(FI), while the second row shows the proportion of sqrt(FI) variance explained by additive genetic (A), dominance genetic (D) and unique environmental (E) factors, with changes in BMI and education. Variance estimates of moderation by BMI were obtained from the full ADE bivariate moderation model between sqrt(FI) and BMI; while variance estimates of moderation by education were obtained from the ADE extended univariate moderation model between sqrt(FI) and education. Quantitative sex-differences were allowed in the models to obtain estimates separately for men and women. Models were adjusted for age.

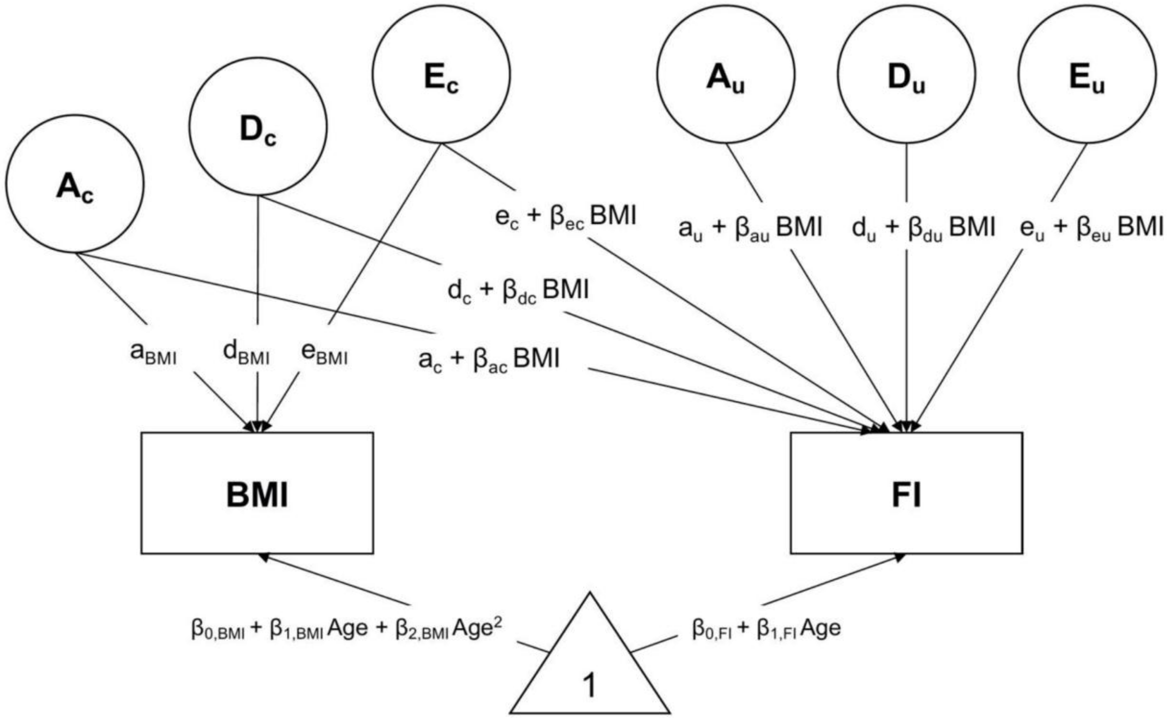

Supplementary Figure 4. Full bivariate moderation model between frailty index (FI) and body mass index (BMI) (for one twin). A, additive genetic factors; D, dominance genetic factors; E, unique environmental factors. AC, DC and EC indicate genetic and environmental influences common to FI and BMI; while AU, DU and EU indicate genetic and environmental influences unique to FI. Total variance of FI by BMI is the sum of common and unique variance estimates; for example, the total additive genetic variance components of FI by BMI can be calculated as: (ac + βac BMI)2 + (au + βau BMI)2.

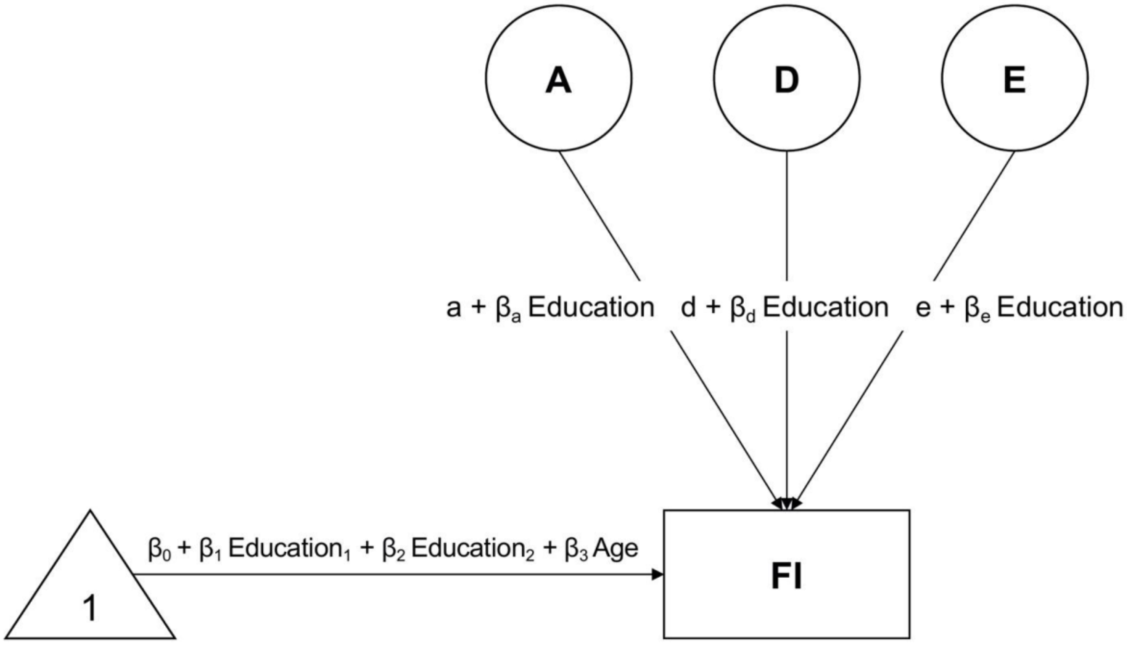

Supplementary Figure 5. Extended univariate moderation model between frailty index (FI) and education (for one twin). A, additive genetic factors; D, dominance genetic factors; E, unique environmental factors. Mean of FI is adjusted for the moderator (i.e. education) of both the individual and his/her co-twin.

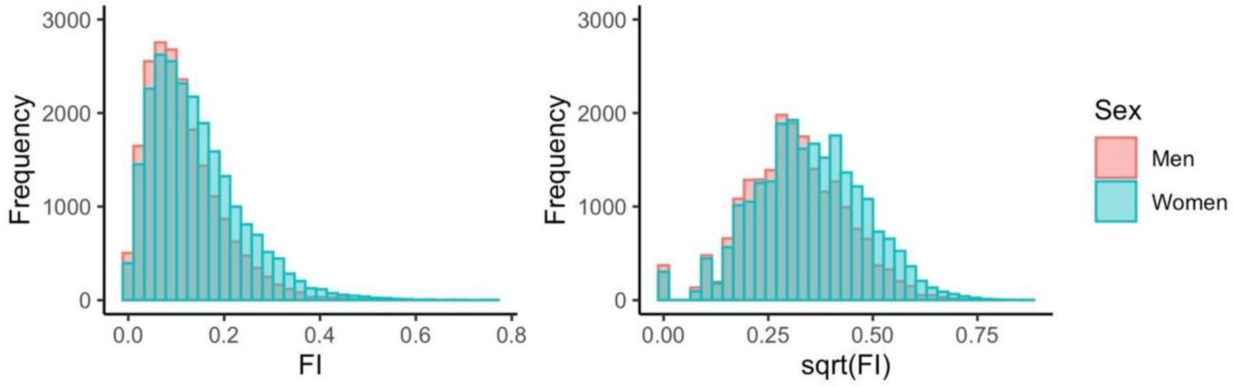

Supplementary Figure 6. Distribution of frailty index (FI) among men and women (n = 42,994). Left panel shows the distribution of the untransformed FI, while right panel shows the distribution of the square-root transformed FI. Red color indicates men, while green color indicates women.
