# Supplementary material for: Sex differences in genetic and environmental influences on frailty and its relation to body mass index and education
Source: Aging (Albany NY). 2021 Jul 6;13(13):16990–7023. doi: 10.18632/aging.203262 (PMC8312411; doi:10.18632/aging.203262)
Supplement: Supplementary Tables [file aging-13-203262-s002.pdf]

SUPPLEMENTARY TABLES

Supplementary Table 1. List of the 44 frailty items and the scoring used for construction of frailty index (FI).

No.	Frailty item	Scoring
1	General health status	Excellent=0; Good=0.25; Average=0.5; Not so good=0.75; Bad=1
2	Health status prevents from doing things normally would like to do	Not at all=0; To some extent=0.5; A great deal=1
3	Serious infections per year (other than respiratory)	0-1 time=0; 2-5 times=0.5; ≥5 times=1
4	Buzzing in ears	No=0; One ear/ both ears=1
5	Angina pectoris	No=0; Yes=1
6	Heart attack	No=0; Yes=1
7	Heart failure	No=0; Yes=1
8	High blood pressure	No=0; Yes=1
9	Lipid disorder (e.g. high cholesterol, high triglycerides)	No=0; Yes=1
10	Vascular spasm in legs (intermittent claudication)	No=0; Yes=1
11	Clot in leg (venous thrombosis)	No=0; Yes=1
12	Cerebral hemorrhage or clot in brain (stroke)	No=0; Yes=1
13	TIA attacks (temporary weakness, paralysis or reduction of sensibility)	No=0; Yes=1
14	Irregular cardiac rhythm/ atrial fibrillation	No=0; Yes=1
15	Chronic lung disease (incl. chronic bronchitis, emphysema)	No=0; Yes=1
16	Dizziness	No=0; Yes=1
17	Rheumatoid arthritis	No=0; Yes=1
18	Knee joint problem	No=0; Yes=1
19	Sciatica	No=0; Yes=1
20	Osteoporosis	No=0; Yes=1
21	Hip joint problem	No=0; Yes=1
22	Back pain	No=0; Yes=1
23	Neck pain	No=0; Yes=1
24	Diabetes (incl. old age diabetes; excl. pregnancy diabetes)	No=0; Yes=1
25	Goiter	No=0; Yes=1
26	Glandular diseases (excl. goiter)	No=0; Yes=1
27	Gall bladder problem	No=0; Yes=1
28	Liver disease (e.g. cirrhosis)	No=0; Yes=1
29	Gout	No=0; Yes=1
30	Kidney disease	No=0; Yes=1
31	Stomach or intestine problems	No=0; Yes=1
32	Recurring urinary tract problems	No=0; Yes=1
33	Cancer, tumor disease or leukemia	No=0; Yes=1
34	Migraine	No=0; Yes=1
35	Asthma	No=0; Yes=1
36	Allergy	No=0; Yes=1
37	Recurrent periods of coughing	No=0; Yes=1
38	Feeling depressed during the past week	Never/ almost never=0; Seldom=0.5; Often/ always/ almost always=1
39	Feeling happy during the past week	Never/ almost never=0; Seldom=0.5; Often/ always/ almost always=1
40	Feeling lonely during the past week	Never/ almost never=0; Seldom=0.5; Often/ always/ almost always=1
41	Physical handicap	No=0; Yes=1
42	Crohn's disease or ulcerative colitis	No=0; Yes=1
43	Vision	Good=0; Reduced=0.5; Highly reduced/ blind=1
44	Hearing	Good=0; Reduced=0.5; Highly reduced=1

Supplementary Table 2. Unadjusted phenotypic correlations, intraclass correlations and cross-twin cross-trait correlations for frailty index (FI), body mass index (BMI) and education.

Zygosity	Phenotypic correlations		Intraclass correlations			Cross-twin cross-trait correlations	
	FI and BMI	FI and Education	FI	BMI	Education	FI and BMI	FI and Education
Total	0.13 (0.12, 0.14)	-0.17 (-0.18, -0.16)					
MZ	0.12 (0.09, 0.14)	-0.17 (-0.19, -0.15)	0.53 (0.51, 0.56)	0.69 (0.67, 0.70)	0.71 (0.69, 0.72)	0.09 (0.07, 0.12)	-0.16 (-0.18, -0.13)
DZ	0.14 (0.13, 0.15)	-0.17 (-0.18, -0.16)	0.23 (0.21, 0.25)	0.24 (0.22, 0.26)	0.50 (0.49, 0.52)	0.07 (0.06, 0.08)	-0.16 (-0.17, -0.15)
MZ males	0.11 (0.08, 0.14)	-0.17 (-0.20, -0.14)	0.47 (0.43, 0.51)	0.66 (0.63, 0.68)	0.69 (0.67, 0.72)	0.09 (0.05, 0.12)	-0.14 (-0.17, -0.10)
MZ females	0.16 (0.13, 0.19)	-0.18 (-0.21, -0.15)	0.55 (0.52, 0.58)	0.69 (0.67, 0.71)	0.72 (0.70, 0.74)	0.14 (0.11, 0.17)	-0.17 (-0.20, -0.14)
DZ males	0.10 (0.07, 0.12)	-0.17 (-0.19, -0.15)	0.19 (0.15, 0.23)	0.27 (0.24, 0.31)	0.50 (0.47, 0.53)	0.03 (0.00, 0.06)	-0.15 (-0.18, -0.13)
DZ females	0.20 (0.18, 0.22)	-0.17 (-0.19, -0.15)	0.28 (0.25, 0.31)	0.32 (0.29, 0.35)	0.57 (0.55, 0.59)	0.12 (0.09, 0.14)	-0.16 (-0.19, -0.14)
DZ opposite-sex	0.10 (0.08, 0.13)	-0.17 (-0.19, -0.15)	0.21 (0.18, 0.23)	0.18 (0.16, 0.21)	0.47 (0.45, 0.49)	0.03 (0.00, 0.06)	-0.14 (-0.17, -0.12)

Note: MZ, monozygotic twins; DZ, dizygotic twins. Phenotypic correlations are the within-individual correlations between FI and BMI, and between FI and education. Intraclass correlations indicate the extent to which each trait correlates within twin pairs. Cross-twin cross-trait correlations show the extent to which FI of the first twin correlate with the other trait (i.e. BMI or education) of the second twin. 95% confidence intervals are presented in parentheses.

Supplementary Table 3. Model fitting results from bivariate models of frailty index (FI) with body mass index (BMI) and education.

Model	-2LL	df	AIC	ΔLL	Δdf	*p*
Bivariate FI and BMI						
Saturated	203422.9	53568	96287	-	-	-
ACE bivariate						
Quantitative sex-limitation	203503.7	53602	96300	80.8	34	$1.12x10^{-5}$
No sex difference	203548.6	53609	96331	125.6	41	$1.60x10^{-10}$
**ADE bivariate**						
**Quantitative sex-limitation**	**203459.9**	**53602**	**96256**	**36.9**	**34**	**0.335**
No sex difference	203485.9	53605	96276	63.0	37	$4.90x10^{-3}$
AE bivariate						
Quantitative sex-limitation	203503.7	53608	96288	80.8	40	$1.43x10^{-4}$
No sex difference	203548.6	53612	96325	125.6	44	$8.73x10^{-10}$
Bivariate FI and education						
Saturated	198109.0	54150	89809	-	-	-
**ACE bivariate**						
**Quantitative sex-limitation**	**198175.0**	**54182**	**89811**	**65.9**	**32**	$\mathbf{3.84x10^{-4}}$
No sex difference	198208.4	54189	89830	99.4	39	$3.53x10^{-7}$
ADE bivariate						
Quantitative sex-limitation	198300.4	54182	89936	191.4	32	$1.29x10^{-24}$
No sex difference	198315.7	54185	89946	206.7	35	$3.18x10^{-26}$
AE bivariate						
Quantitative sex-limitation	198328.3	54188	89952	219.3	38	$2.39x10^{-27}$
No sex difference	198360.8	54192	89977	251.8	42	$1.02x10^{-31}$

Note: AIC, Akaike's Information Criterion; LL, log-likelihood; df, degrees of freedom; *p*, *p*-values of likelihood ratio tests compared with the saturated models. Opposite-sex twins were excluded in the bivariate analyses. All bivariate models between FI and education had significant worse model fit than the saturated model, since an ADE model fits better for FI while an ACE model fits better for education. During assumption testing, equating means of education across zygosity resulted in a significantly worse fit of data compared to the saturated model; therefore, means of education were estimated separately across zygosity in bivariate models between FI and education. All models were adjusted for age (as linear effect for FI, and linear+quadratic effect for BMI and education). Best-fitting models are shown in bold, and the parameter estimates of these models are presented in Supplementary Table 4.

Supplementary Table 4. Parameter estimates (95% CI) from the best-fitting bivariate models.

Model	Variance components				Genetic and environmental correlations				Bivariate heritability			
	A	D/C	H	E	r_A	r_D / r_C	r_H	r_E	Bivariate A	Bivariate D/C	Bivariate H	Bivariate E
**ADE bivariate model between FI and BMI**												
FI	M: 6% (0, 22)	M: 38% (21, 54)	M: 44% (40, 48)	F: 56% (52, 60)								
	F: 42% (28, 55)	F: 11% (0, 25)	F: 53% (50, 55)	F: 47% (45, 50)								
BMI	M: 41% (27, 55)	M: 25% (10, 39)	M: 66% (64, 68)	M: 34% (32, 36)	M: 0.61 (-0.25, 1.46)	M: 0.01 (-0.33, 0.36)	M: 0.19 (0.14, 0.23)	M: 0.05 (0.01, 0.10)	M: 78% (-3, 159)	M: 3% (-82, 88)	M: 81% (65, 97)	M: 19% (3, 35)
	F: 56% (42, 69)	F: 13% (0, 27)	F: 69% (67, 71)	F: 31% (29, 33)	F: 0.48 (0.29, 0.67)	F: -0.64 (-1.70, 0.42)	F: 0.26 (0.22, 0.29)	F: 0.06 (0.02, 0.10)	F: 130% (79, 181)	F: -43% (-96, 10)	F: 87% (78, 95)	F: 13% (5, 22)
**ACE bivariate model between FI and education**												
FI	M: 39% (35, 43)	M: 1% (0, 3)	M: 39% (35, 43)	M: 60% (56, 63)								
	F: 51% (48, 54)	F: 1% (0, 3)	F: 51% (48, 54)	F: 48% (46, 51)								
Education	M: 41% (34, 48)	M: 25% (19, 31)	M: 41% (34, 48)	M: 35% (32, 37)	M: -0.02 (-0.15, 0.11)	M: -1.00 (-1.00, -1.00)	M: -0.02 (-0.15, 0.11)	M: -0.06 (-0.10, -0.01)	M: 8% (-46, 61)	M: 65% (23, 107)	M: 8% (-46, 61)	M: 28% (6, 49)
	F: 35% (29, 42)	F: 29% (23, 34)	F: 35% (29, 42)	F: 36% (34, 38)	F: -0.04 (-0.15, 0.07)	F: -1.00 (-1.00, -1.00)	F: -0.04 (-0.15, 0.07)	F: -0.01 (-0.05, 0.03)	F: 22% (-40, 84)	F: 74% (22, 126)	F: 22% (-40, 84)	F: 4% (-17, 25)

Note: BMI, body mass index; FI, frailty index; CI, Wald-type confidence interval; A, additive genetic factors; D, dominance genetic factors; H, total genetic factors/ broad-sense heritability; C, common environmental factors; E, unique environmental factors; *r*, correlation between variance components. M and F represents parameter estimates for men and women respectively. Bivariate heritability is the proportion of phenotypic correlation explained by genetic and environmental factors.

Supplementary Table 5. Model fitting results from moderation models of frailty index (FI) by body mass index (BMI).

Model	-2LL	df	AIC	Comp	ΔLL	Δdf	*p*
ACE bivariate							
1. Full moderation	146418.0	38963	68492	-	-	-	-
**ADE bivariate**							
**2. Full moderation**	**146354.9**	**38963**	**68429**	**-**	**-**	**-**	**-**
3. Drop all covariance moderation	146453.0	38969	68515	2	98.1	6	$6.29x10^{-19}$
4. Drop all moderation	146653.5	38975	68704	2	298.7	12	$9.01x10^{-57}$
AE bivariate							
5. Full moderation	146489.1	38973	68543	2	134.2	10	$6.34x10^{-24}$
6. Drop all covariance moderation	146615.8	38977	68662	5	126.7	4	$1.96x10^{-26}$
7. Drop all moderation	146697.7	38981	68736	5	208.6	8	$9.73x10^{-41}$

Note: AIC, Akaike's Information Criterion; Comp, model of comparison; df, degrees of freedom; LL, Log-likelihood; *p*, *p*-values of likelihood ratio tests compared with the models of comparison. Opposite-sex twins were excluded in the models. Quantitative sex differences were allowed to obtain separate estimates for men and women. All models were adjusted for age (as linear effect for FI, and linear+quadratic effect for BMI). Best-fitting model is shown in bold.

Supplementary Table 6. Model fitting results from moderation models of frailty index (FI) by education.

Models	-2LL	df	AIC	Comp	ΔLL	Δdf	*p*
ACE bivariate							
1. Full moderation	145282.4	40231	64820	-	-	-	-
2. Drop all covariance moderation	145289.7	40237	64816	1	7.3	6	0.297
3. Drop all moderation	145537.7	40243	65052	1	255.3	12	1.09×10^{-47}
ADE bivariate							
4. Full moderation	145418.6	40231	64957	-	-	-	-
5. Drop all covariance moderation	145423.2	40237	64949	4	4.6	6	0.592
6. Drop all moderation	145665.6	40243	40243	4	247.0	12	5.71×10^{-46}
AE bivariate							
7. Full moderation	145450.9	40241	64969	1	168.4	10	5.92×10^{-31}
8. Drop all covariance moderation	145454.7	40245	64965	7	3.8	4	0.432
9. Drop all moderation	145694.2	40249	65196	7	243.3	8	4.42×10^{-48}
ACE extended univariate							
10. Full moderation	47816.3	20109	7598	-	-	-	-
11. Drop all moderation	48064.9	20115	7835	10	248.6	6	8.24×10^{-51}
**ADE extended univariate**							
**12. Full moderation**	**47793.4**	**20109**	**7575**	**-**	**-**	**-**	**-**
13. Drop all moderation	48039.4	20115	7809	12	246.0	6	2.90×10^{-50}
AE extended univariate							
14. Full moderation	47823.0	20113	7597	12	29.6	-	-
15. Drop all moderation	48064.9	20117	7831	14	241.9	4	3.69×10^{-51}

Note: AIC, Akaike's Information Criterion; Comp, model of comparison; df, degrees of freedom; LL, log-likelihood; *p*, *p*-values of likelihood ratio tests compared with the models of comparison. Opposite-sex twins were excluded in the models. Quantitative sex differences were allowed to obtain separate estimates for men and women. Bivariate models were adjusted for age (as linear effect for FI, and linear+quadratic effect for education); extended univariate models were adjusted for age, as well as education for both the individual and the co-twin. Best-fitting model is shown in bold. Due to the non-significant moderation on the covariance between FI and education in the bivariate models, the more parsimonious ADE extended univariate model was used.

Supplementary Table 7. Model fitting results and parameter estimates from univariate sex-limitation models of the square-root transformed frailty index [sqrt(FI)].

Model	Model fit statistics				Parameter estimates for men and women (95% CI)				
	AIC	ΔLL	Δdf	*p*	A	D/C	H	E	r_{fm}
Saturated	-146609	-	-	-	-	-	-	-	-
ADE full sex-limitation	-146620	20.6	16	0.196	M: 7% (0, 23)<br>F: 40% (27, 53)	M: 34% (17, 50)<br>F: 10% (0, 24)	M: 41% (38, 45)<br>F: 50% (47, 53)	M: 59% (55, 62)<br>F: 50% (47, 53)	0.68 (0.49, 0.93)
**ADE quantitative sex-limitation**	**-146621**	**21.4**	**17**	**0.208**	**M: 0% (0, 1)**<br>**F: 40% (26, 53)**	**M: 41% (38, 45)**<br>**F: 10% (0, 24)**	**M: 42% (38, 45)**<br>**F: 50% (47, 53)**	**M: 58% (55, 62)**<br>**F: 50% (47, 53)**	**1.00 (NA)**
ADE no sex difference	-146609	35.2	18	0.009	M: 0% (0, 3)<br>F: 43% (29, 57)	M: 46% (41, 50)<br>F: 3% (0, 17)	M: 46% (44, 48)<br>F: 46% (44, 48)	M: 54% (52, 56)<br>F: 54% (52, 56)	1.00 (NA)
ACE full sex-limitation	-146602	38.7	16	0.001	M: 38% (34, 41)<br>F: 49% (46, 52)	M: 0% (0, 0)<br>F: 0% (0, 0)	M: 38% (34, 41)<br>F: 49% (46, 52)	M: 62% (59, 66)<br>F: 51% (48, 54)	0.77 (0.65, 0.90)
AE full sex-limitation	-146606	38.7	18	0.003	M: 38% (34, 41)<br>F: 49% (46, 52)	M: 0% (NA)<br>F: 0% (NA)	M: 38% (34, 41)<br>F: 49% (46, 52)	M: 62% (59, 66)<br>F: 51% (48, 54)	0.77 (0.65, 0.90)

Note: AIC, Akaike's Information Criterion; LL, log-likelihood; df, degrees of freedom; *p*, *p*-values of likelihood ratio tests compared with the saturated model. CIs are Wald-type confidence intervals with lower and upper bounds of 0 and 1. A, additive genetic factors; D, dominance genetic factors; C, common environmental factors; H, total genetic factors/ broad-sense heritability; E, unique environmental factors; r_{fm}, genetic correlation between men and women, estimated using opposite-sex twins. M and F represents parameter estimates for men and women respectively. Full sex-limitation models allowed both quantitative and qualitative sex differences. In ADE quantitative sex-limitation model, r_{fm} was fixed to be 1. In ADE no sex difference model, broad-sense heritability of men and women were equated, but variance difference between sex was allowed. ACE and AE sub-models are not shown as the full models fit significantly worse than the saturated model. All models were adjusted for age. Best-fitting model is shown in bold.

Supplementary Table 8. Model fitting results and parameter estimates from univariate sex-limitation models of the frailty index (FI) using "direct symmetric approach.

Model	Model fit statistics				Parameter estimates for men and women (95% CI)				
	AIC	ΔLL	Δdf	*p*	A	D/C	H	E	r_{fm}
Saturated	19953	-	-	-	-	-	-	-	-
ADE full sex-limitation	19940	19.1	16	0.264	M: 7% (-10, 23)<br>F: 41% (28, 55)	M: 38% (20, 55)<br>F: 11% (-3, 25)	M: 44% (41, 48)<br>F: 52% (50, 55)	M: 56% (52, 59)<br>F: 48% (45, 50)	0.69 (0.41, 0.96)
**ADE quantitative sex-limitation**	**19939**	**19.7**	**17**	**0.288**	**M: 0% (0, 1)**<br>**F: 41% (28, 55)**	**M: 44% (41, 48)**<br>**F: 11% (-3, 25)**	**M: 45% (41, 48)**<br>**F: 52% (50, 55)**	**M: 55% (52, 59)**<br>**F: 48% (45, 50)**	**1.00 (NA)**
ADE no sex difference	19949	32.1	18	0.021	M: 0% (-1, 2)<br>F: 44% (31, 58)	M: 49% (45, 52)<br>F: 4% (-10, 19)	M: 49% (47, 51)<br>F: 49% (47, 51)	M: 51% (49, 53)<br>F: 51% (49, 53)	1.00 (NA)
ACE full sex-limitation*	19940	19.1	16	0.264	M: 63% (53, 74)<br>F: 58% (49, 66)	M: -19% (-27, -10)<br>F: -5% (-13, 2)	M: 63% (53, 74)<br>F: 51% (49, 54)	M: 56% (52, 59)<br>F: 48% (45, 50)	0.24 (-0.03, 0.51)
AE full sex-limitation	19957	40.4	18	0.002	M: 41% (37, 44)<br>F: 51% (49, 54)	M: 0% (NA)<br>F: 0% (NA)	M: 41% (37, 44)<br>F: 51% (49, 54)	M: 59% (56, 63)<br>F: 49% (46, 51)	0.76 (0.64, 0.88)

Note: AIC, Akaike's Information Criterion; LL, log-likelihood; df, degrees of freedom; *p*, *p*-values of likelihood ratio tests compared with the saturated model. CIs are Wald-type confidence intervals. Variance component estimates are allowed to be negative in the "direct symmetric approach" (Verhulst *et al.* 2019). A, additive genetic factors; D, dominance genetic factors; C, common environmental factors; H, total genetic factors/ broad-sense heritability; E, unique environmental factors; r_{fm}, genetic correlation between men and women, estimated using opposite-sex twins. M and F represents parameter estimates for men and women respectively. Full sex-limitation models allowed both quantitative and qualitative sex differences. In ADE quantitative sex-limitation model, r_{fm} was fixed to be 1. In ADE no sex difference model, broad-sense heritability of men and women were equated, but variance difference between sex was allowed. All models were adjusted for age. Best-fitting model is shown in bold.

* An ACE quantitative sex-limitation model cannot be fitted due to an issue with the expected opposite sex twin covariance of √(VCm*VCf) (using notation from Verhulst *et al.*) when one common environmental component was negative, a problem identified in Verhulst *et al.* (2019).

Supplementary Table 9. Model fitting results from bivariate models of frailty index (FI) with body mass index (BMI) and education using "direct symmetric approach.

Model	-2LL	df	AIC	ΔLL	Δdf	*p*
Bivariate FI and BMI						
Saturated	203422.9	53568	96287	-	-	-
**ACE bivariate**						
**Quantitative sex-limitation**	**203459.9**	**53602**	**96256**	**36.9**	**34**	**0.335**
No sex difference	203510.4	53609	96292	87.5	41	3.26×10^{-5}
**ADE bivariate**						
**Quantitative sex-limitation**	**203459.9**	**53602**	**96256**	**36.9**	**34**	**0.335**
No sex difference	203485.1	53605	96276	62.1	37	5.96×10^{-3}
AE bivariate						
Quantitative sex-limitation	203503.7	53608	96288	80.8	40	1.43×10^{-4}
No sex difference	203548.6	53612	96325	125.6	44	8.72×10^{-10}
Bivariate FI and education						
Saturated	198109.0	54150	89809	-	-	-
**ACE bivariate**						
**Quantitative sex-limitation**	**198147.1**	**54182**	**89783**	**38.1**	**32**	**0.213**
No sex difference	198186.2	54189	89808	77.2	39	2.60×10^{-4}
**ADE bivariate**						
**Quantitative sex-limitation**	**198147.1**	**54182**	**89783**	**38.1**	**32**	**0.213**
No sex difference	198162.6	54185	89793	53.6	35	0.023
AE bivariate						
Quantitative sex-limitation	198328.3	54188	89952	219.3	38	2.39×10^{-27}
No sex difference	198360.8	54192	89977	251.8	42	1.02×10^{-31}

Note: AIC, Akaike's Information Criterion; LL, log-likelihood; df, degrees of freedom; *p*, *p*-values of likelihood ratio tests compared with the saturated models. Variance component estimates are allowed to be negative in the "direct symmetric approach" (Verhulst *et al.* 2019). Opposite-sex twins were excluded in the bivariate analyses. During assumption testing, equating means of education across zygosity resulted in a significantly worse fit of data compared to the saturated model; therefore, means of education were estimated separately across zygosity in bivariate models between FI and education. All models were adjusted for age (as linear effect for FI, and linear+quadratic effect for BMI and education). Best-fitting models are shown in bold, and the parameter estimates of these models are presented in Supplementary Table 10.

Supplementary Table 10. Parameter estimates (95% CI) from the best-fitting bivariate models using "direct symmetric approach".

Model	Variance components				Genetic and environmental correlations				Bivariate heritability			
	A	D/C	H	E	r_A	r_D / r_C	r_H	r_E	Bivariate A	Bivariate D/C	Bivariate H	Bivariate E
**ACE bivariate model between FI and BMI †**												
FI	M: 63% (52, 73)	M: -19% (-27, -10)	M: 63% (52, 73)	F: 56% (52, 60)								
	F: 58% (50, 67)	F: -5% (-13, 2)	F: 58% (50, 67)	F: 47% (45, 50)								
BMI	M: 78% (70, 86)	M: -12% (-19, -5)	M: 78% (70, 86)	M: 34% (34, 36)	M: 0.15 (0.05, 0.24)	M: N/A*	M: 0.15 (0.05, 0.24)	M: 0.05 (0.01, 0.10)	M: 82% (30, 135)	M: -1% (-46, 43)	M: 82% (30, 135)	M: 19% (3, 35)
	F: 75% (68, 82)	F: -7% (-13, 0)	F: 75% (68, 82)	F: 31% (29, 33)	F: 0.18 (0.09, 0.26)	F: N/A*	F: 0.18 (0.09, 0.26)	F: 0.06 (0.02, 0.10)	F: 65% (34, 97)	F: 22% (-6, 49)	F: 65% (34, 97)	F: 13% (5, 22)
**ADE bivariate model between FI and BMI †**												
FI	M: 6% (-10, 22)	M: 38% (21, 55)	M: 44% (40, 48)	F: 56% (52, 60)								
	F: 42% (28, 55)	F: 11% (-3, 25)	F: 53% (50, 55)	F: 47% (45, 50)								
BMI	M: 41% (28, 55)	M: 25% (11, 39)	M: 66% (64, 68)	M: 34% (32, 36)	M: 0.61 (-0.34, 1.56)	M: 0.01 (-0.36, 0.38)	M: 0.19 (0.14, 0.23)	M: 0.05 (0.01, 0.10)	M: 78% (-8, 165)	M: 3% (-88, 94)	M: 81% (65, 97)	M: 19% (3, 35)
	F: 56% (44, 67)	F: 13% (2, 25)	F: 69% (67, 71)	F: 31% (29, 33)	F: 0.48 (0.28, 0.68)	F: -0.64 (-1.71, 0.43)	F: 0.26 (0.22, 0.29)	F: 0.06 (0.02, 0.10)	F: 130% (76, 184)	F: -43% (-99, 13)	F: 87% (78, 95)	F: 13% (5, 22)
**ACE bivariate model between FI and education ‡**												
FI	M: 63% (52, 73)	M: -19% (-27, -10)	M: 63% (52, 73)	M: 56% (52, 59)								
	F: 58% (49, 66)	F: -5% (-12, 2)	F: 58% (49, 66)	F: 47% (45, 50)								
Education	M: 41% (34, 48)	M: 24% (18, 30)	M: 41% (34, 48)	M: 35% (32, 37)	M: 0.05 (-0.08, 0.17)	M: N/A*	M: 0.05 (-0.08, 0.17)	M: -0.07 (-0.11, 0.02)	M: -24% (-91, 43)	M: 92% (36, 148)	M: -24% (-91, 43)	M: 32% (11, 53)
	F: 36% (29, 42)	F: 28% (23, 34)	F: 36% (29, 42)	F: 36% (34, 38)	F: -0.02 (-0.13, 0.10)	F: N/A*	F: -0.02 (-0.13, 0.10)	F: -0.01 (-0.05, 0.03)	F: 11% (-58, 79)	F: 84% (26, 142)	F: 11% (-58, 79)	F: 5% (-16, 27)
**ADE bivariate model between FI and education ‡**												
FI	M: 6% (-10, 22)	M: 38% (20, 55)	M: 44% (41, 48)	M: 56% (52, 59)								
	F: 42% (29, 56)	F: 10% (-4, 24)	F: 53% (50, 55)	F: 47% (45, 50)								
Education	M: 114% (102, 125)	M: -48% (-61, -36)	M: 65% (63, 68)	M: 35% (32, 37)	M: -0.89 (-2.02, 0.24)	M: N/A*	M: -0.12 (-0.17, -0.07)	M: -0.07 (-0.11, -0.02)	M: 252% (146, 358)	M: -184% (-297, -72)	M: 68% (47, 89)	M: 32% (11, 53)
	F: 121% (111, 131)	F: -57% (-67, -46)	F: 64% (62, 66)	F: 36% (34, 38)	F: -0.28 (-0.41, -0.15)	F: N/A*	F: -0.12 (-0.16, -0.09)	F: -0.01 (-0.05, 0.03)	F: 262% (152, 372)	F: -168% (-283, -52)	F: 95% (73, 116)	F: 5% (-16, 27)

Note: BMI, body mass index; FI, frailty index; CI, Wald-type confidence interval; A, additive genetic factors; D, dominance genetic factors; H, total genetic factors/ broad-sense heritability; C, common environmental factors; E, unique environmental factors; *r*, correlation between variance components. M and F represents parameter estimates for men and women respectively. Bivariate heritability is the proportion of phenotypic correlation explained by genetic and environmental factors. Variance component estimates are allowed to be negative in the "direct symmetric approach" (Verhulst *et al.* 2019).

* Correlation between C or D components were not defined when the variance components are negative.

† The same conclusion can be drawn from either the ACE and ADE bivariate models between FI and BMI, in which there is a modest genetic correlation (r_H) between FI and BMI, and their phenotypic correlation is mostly explained by genetic factors (bivariate H).

‡ Although both ACE and ADE bivariate models have the same model fit when allowing for negative variances, the ACE bivariate model is more in line with the observed cross-twin cross-trait correlations (MZ twins: -0.07; DZ twins: -0.08, shown in Table 2) that the phenotypic correlation between FI and education is largely explained by common environmental factors (bivariate C = 92% in men and 84% in women, slightly more than 65% and 74% using the standard approach). In contrast, the solution in the ADE bivariate model yielded a highly negative and non-sensical D component, and it causes all the common environmental influences on the covariance going to genetic factors – a result not in line with the standard interpretations of the observed cross-twin cross-trait correlations.
