# Supplementary material for: Sex differences in genetic and environmental influences on frailty and its relation to body mass index and education
Source: Aging (Albany NY). 2021 Jul 6;13(13):16990–7023. doi: 10.18632/aging.203262 (PMC8312411; doi:10.18632/aging.203262)
Supplement: Supplementary Methods [file aging-13-203262-s003.pdf]

SUPPLEMENTARY METHODS

INTRODUCTION

In this appendix we outline an approach to estimating the genetic correlation between males and females for a phenotype which has genetic contributions both from additive and dominance deviations to its variance. *1.* Using the biometric model in a simplified two-allelic locus with equal allele frequencies, we will derive the resulting correlation between genetic contributions in males and females with different additive and dominance deviation contributions genotypic expression. *2.* We then show that the resulting correlation depends on allele frequencies. *3.* Finally, we suggest an intuitive solution for estimation of the genetic correlation between males and females, and compare its performance with other possible solutions in a series of simulations.

As outlined below, our suggested model for the covariance between twins in opposite sexed DZ pairs is

$$Cov\left(\begin{bmatrix} Y_1 \\ Y_2 \end{bmatrix}\right)_{osDZ} = \begin{bmatrix} \sigma_{Af}^2+\sigma_{Df}^2+\sigma_{Ef}^2 & r_{fm}\left(0.5\sigma_{Af}\sigma_{Am}+0.25\sigma_{Df}\sigma_{Dm}+0.25\sigma_{Af}\sigma_{Dm}+0.25\sigma_{Df}\sigma_{Am}\right) \\ r_{fm}\left(0.5\sigma_{Af}\sigma_{Am}+0.25\sigma_{Df}\sigma_{Dm}+0.25\sigma_{Af}\sigma_{Dm}+0.25\sigma_{Df}\sigma_{Am}\right) & \sigma_{Am}^2+\sigma_{Dm}^2+\sigma_{Em}^2 \end{bmatrix} \quad (1)$$

where σ_X^2 represent contributions to variance and covariance from source X. X are A, additive genetic contributions, D, dominant genetic contributions, and E, individually unique contributions. The sub-indexes are also complemented with f and m to indicate female and male sources. Below we show that this model, although not uniformly unbiased, has some features which makes it suitable for situations where additive and dominance contributions to variance of the phenotype exists in both sexes (possibly in different proportions).

Derivation of correlation due to genetics from biometric model

Following the set-up of the biometric model in Neale and Maes [1] we define one autosomal locus having allele A and a at equal ½ frequencies in a population. We define the genotypic effect on Y to be $-d$ for allele combination aa, h for allele combination Aa, and d for allele combination AA (see Supplementary Figure 7). Note that the definition of genotypic effect is somewhat arbitrary, see e.g. Neale and Maes [1].

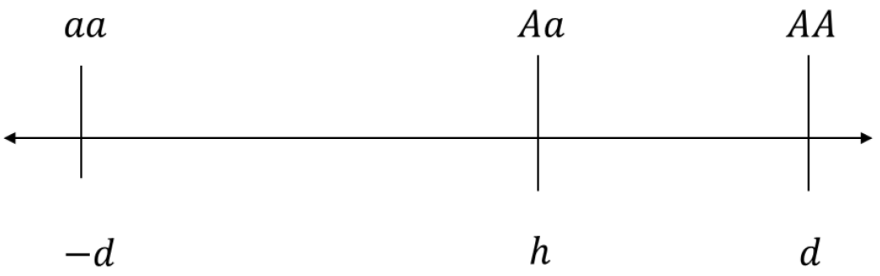

Supplementary Figure 7. Graphical representation of the genotypic effect. (Adapted from Neale and Maes [1]).

Here h indicates the deviation from additivity; we assume h to be bounded between $-d$ and d to keep a biologically feasible interpretation of additivity *vs* dominance/recessiveness. The ½ frequencies means that a random individual has a ¼ probability of having allele combination aa, a ½ probability of having Aa, and a ¼ probability of having AA. The mean genotypic contribution can be written as (using the law of total expectation).

$$\mu_Y = E(Y) = E(Y|aa)\Pr(aa) + E(Y|Aa)\Pr(Aa) + E(Y|AA)\Pr(AA) = -d\cdot\frac{1}{4} + h\cdot\frac{1}{2} + d\cdot\frac{1}{4} = \frac{1}{2}h. \quad (2)$$

The variance can be calculated as

$$\begin{aligned} Var(Y) &= E\left(\left(Y - E(Y)\right)^2\right) = E\left(Y^2\right) - E(Y)^2 \\ &= E\left(Y^2|aa\right)\Pr(aa) + E\left(Y^2|Aa\right)\Pr(Aa) + E\left(Y^2|AA\right)\Pr(AA) - \left(\frac{1}{2}h\right)^2 \\ &= (-d)^2\cdot\frac{1}{4} + h^2\cdot\frac{1}{2} + d^2\cdot\frac{1}{4} - \frac{1}{4}h^2 = \frac{1}{2}d^2 + \frac{1}{4}h^2. \end{aligned} \quad (3)$$

Now, let the same locus contribute to two phenotypes, Y_1 and Y_2 (e.g., same phenotype in males and females), possibly with different additivity and dominance deviation. Define the genotypic effects to be $\{-d_1, h_1, d_1\}$ and $\{-d_2, h_2, d_2\}$ for Y_1 and Y_2, respectively. Using the frequencies of allele combination in full siblings (or DZ twins), following Neale and Maes [1], we can create a table indicating the contributions to the covariance from each combination of allele in the two siblings (Supplementary Table 11).

Supplementary Table 11. Contributions to covariance, and expected frequency, between two DZ twins.

Sibling 1 alleles	Sibling 2 alleles	Genotypic effect minus mean, sibling 1	Genotypic effect minus mean, sibling 2	Contribution to covariance	Frequency
AA	*AA*	$d_1 - \frac{1}{2}h_1$	$d_2 - \frac{1}{2}h_2$	$d_1d_2 - \frac{1}{2}d_1h_2 - \frac{1}{2}h_1d_2 + \frac{1}{4}h_1h_2$	$\frac{9}{64}$
AA	*Aa*	$d_1 - \frac{1}{2}h_1$	$\frac{1}{2}h_2$	$\frac{1}{2}d_1h_2 - \frac{1}{4}h_1h_2$	$\frac{6}{64}$
AA	*aa*	$d_1 - \frac{1}{2}h_1$	$-d_2 - \frac{1}{2}h_2$	$-d_1d_2 - \frac{1}{2}d_1h_2 + \frac{1}{2}h_1d_2 + \frac{1}{4}h_1h_2$	$\frac{1}{64}$
Aa	*AA*	$\frac{1}{2}h_1$	$d_2 - \frac{1}{2}h_2$	$\frac{1}{2}h_1d_2 - \frac{1}{4}h_1h_2$	$\frac{6}{64}$
Aa	*Aa*	$\frac{1}{2}h_1$	$\frac{1}{2}h_2$	$\frac{1}{4}h_1h_2$	$\frac{20}{64}$
Aa	*aa*	$\frac{1}{2}h_1$	$-d_2 - \frac{1}{2}h_2$	$-\frac{1}{2}h_1d_2 - \frac{1}{4}h_1h_2$	$\frac{6}{64}$
aa	*AA*	$-d_1 - \frac{1}{2}h_1$	$d_2 - \frac{1}{2}h_2$	$-d_1d_2 + \frac{1}{2}d_1h_2 - \frac{1}{2}h_1d_2 + \frac{1}{4}h_1h_2$	$\frac{1}{64}$
aa	*Aa*	$-d_1 - \frac{1}{2}h_1$	$\frac{1}{2}h_2$	$-\frac{1}{2}d_1h_2 - \frac{1}{4}h_1h_2$	$\frac{6}{64}$
aa	*aa*	$-d_1 - \frac{1}{2}h_1$	$-d_2 - \frac{1}{2}h_2$	$d_1d_2 + \frac{1}{2}d_1h_2 + \frac{1}{2}h_1d_2 + \frac{1}{4}h_1h_2$	$\frac{9}{64}$

We can thus calculate the contribution to covariance from this locus between phenotypes and between DZ twins as

$$
\begin{aligned}
Cov(Y_1,Y_2) &= \sum E\left(\left(Y_1-\mu_{Y_1}\right)\left(Y_2-\mu_{Y_2}\right)\right)\\
&= \sum E\left(\left(Y_1-\mu_{Y_1}\right)\left(Y_2-\mu_{Y_2}\right) \mid \text{allelic combination}\right)\Pr\left(\text{allelic combination}\right) =\\
&= \frac{9}{64}\left(d_1d_2-\frac{1}{2}d_1h_2-\frac{1}{2}h_1d_2+\frac{1}{4}h_1h_2\right)+\frac{6}{64}\left(\frac{1}{2}d_1h_2-\frac{1}{4}h_1h_2\right)\\
&+\frac{1}{64}\left(-d_1d_2-\frac{1}{2}d_1h_2+\frac{1}{2}h_1d_2+\frac{1}{4}h_1h_2\right)+\frac{6}{64}\left(\frac{1}{2}h_1d_2-\frac{1}{4}h_1h_2\right)+\frac{20}{64}\left(\frac{1}{4}h_1h_2\right)\\
&+\frac{6}{64}\left(-\frac{1}{2}h_1d_2-\frac{1}{4}h_1h_2\right)+\frac{1}{64}\left(-d_1d_2+\frac{1}{2}d_1h_2-\frac{1}{2}h_1d_2+\frac{1}{4}h_1h_2\right)\\
&+\frac{6}{64}\left(-\frac{1}{2}d_1h_2-\frac{1}{4}h_1h_2\right)+\frac{9}{64}\left(d_1d_2+\frac{1}{2}d_1h_2+\frac{1}{2}h_1d_2+\frac{1}{4}h_1h_2\right)\\
&= \frac{1}{64}(9-1-1+9)d_1d_2+\frac{1}{64}\left(-\frac{9}{2}+\frac{6}{2}-\frac{1}{2}+\frac{1}{2}-\frac{6}{2}+\frac{9}{2}\right)d_1h_2\\
&+\frac{1}{64}\left(-\frac{9}{2}+\frac{1}{2}+\frac{6}{2}-\frac{6}{2}-\frac{1}{2}+\frac{9}{2}\right)h_1d_2+\frac{1}{64}\left(\frac{9}{4}-\frac{6}{4}+\frac{1}{4}-\frac{6}{4}+\frac{20}{4}-\frac{6}{4}+\frac{1}{4}-\frac{6}{4}+\frac{9}{4}\right)h_1h_2\\
&= \frac{1}{64}\cdot 16\cdot d_1d_2+0\cdot d_1h_2+0\cdot h_1d_2+\frac{1}{64}\cdot\frac{16}{4}\cdot h_1h_2=\frac{1}{4}d_1d_2+\frac{1}{16}h_1h_2
\end{aligned}
\tag{4}
$$

And thus, the expected correlation in this locus is

$$
Cor(Y_1,Y_2)=\frac{Cov(Y_1,Y_2)}{\sqrt{Var(Y_1)}\sqrt{Var(Y_2)}}=\frac{\frac{1}{4}d_1d_2+\frac{1}{16}h_1h_2}{\sqrt{\frac{1}{2}d_1^2+\frac{1}{4}h_1^2}\sqrt{\frac{1}{2}d_2^2+\frac{1}{4}h_2^2}}
\tag{5}
$$

We may investigate how the correlation will depend on dominance deviations by varying the *h*'s between + and – the *d*'s. In Supplementary Figure 8 we have plotted expected correlations over varying degrees of dominance deviations in the two genotypic effect for the different phenotypes, the *d*'s have value 1. An assumption is that d_1 and d_2 have same sign, thus the correlation is positive.

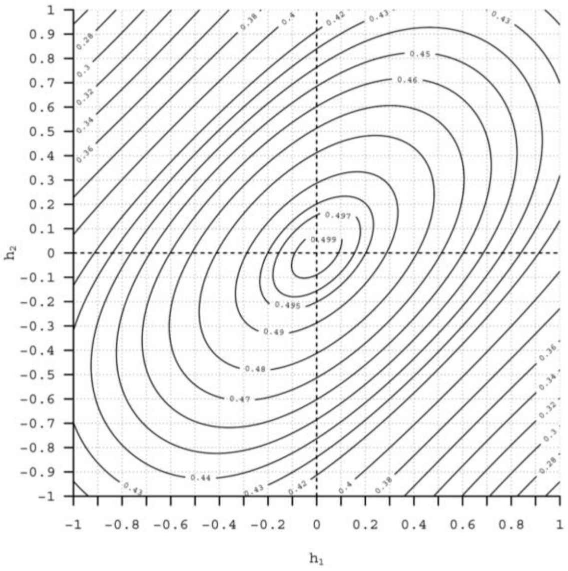

Supplementary Figure 8. Contour plot of deviations from additivity, when *h*'s are at ±1 there is maximal dominance deviation.

From the derivation and plot we may notice several things: 1. When one genotypic effect is at maximum dominance, and the other at perfect additivity, the correlation is $\frac{1}{\sqrt{6}} \approx 0.4082$. 2. When both genotypic effects have maximum deviation, in the same ± direction, the correlation is $\frac{5}{12} \approx 0.4167$. 3. When both genotypic effects are purely additive the correlation is 0.5. 4. When the genotypic effects are at maximum deviation, but in opposite ± direction, the correlation is 0.25.

Thus, the contribution to correlation between two phenotypes in different DZ twins depends on the amount and direction (i.e., dominance or recessiveness) of dominance deviation.

The correlation depends on the allele frequencies

If we want to expand on previous simplified equal allele frequency and allow any allele frequency, we quickly generate a complex expression. Therefore, we set up a simulation with a larger number of alleles and a large number of DZ twins, where we vary the dominance deviations, to assess the impact of varying allele frequencies.

We simulated 100,000 DZ pairs and 100 assumed contributing loci under different scenarios. In all scenarios we assumed that the sign and maximal genotypic effect were the same for both phenotypes. Additional assumptions were non-assortative mating (i.e., parents were treated as two random individuals in the population), no association between minor allele frequency (MAF) and genotypic effect, no interactions between loci, and no interactions with any ‘environmental’ (i.e., non-genetic) variable. The genotypic effects were drawn randomly from a standard normal distribution. Each scenario is based on different MAF;

1. MAF = 0.5 for all loci.

2. MAF drawn from a uniform distribution between 0 and 0.5.

3. MAF drawn from a uniform distribution between 0 and 0.1.

4. MAF drawn from a random distribution between 0 and 0.5 with an ‘L’-shape (a situation with MAF pushed towards 0 but still covering the full range).

The MAF distribution in point 4 is presented in Supplementary Figure 9, and was found through

$$MAF = \frac{e^{U^2} - 1}{2\left(e^{0.25} - 1\right)}, \text{where } U \sim Uniform(0,.5) \qquad (6)$$

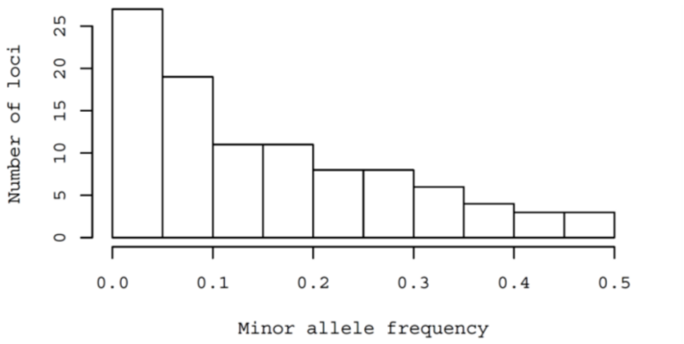

Supplementary Figure 9. A specific scenario for minor allele frequency distribution.

From the each of the four simulations with different MAF distributions we produce three separate series of estimates over varying dominance deviation: scenario A, varying degree of dominance deviation in one phenotype, and fixed additive genotypic effect in other phenotype, scenario B, varying degree of dominance deviation in one phenotype, and maximal dominance deviation genotypic effect in other, and Scenario C, varying dominance deviation vs maximal recessive deviance. Note that dominance in the *minor* allele corresponds to recessiveness in the *major* allele, and *vice versa*. These simulations serve to investigate the impact of MAF on correlation due to genetic effects between DZ twins (and full siblings) in the scenario where genotypic expression with regard to dominance deviations differ between the twins, results are presented in Supplementary Figure 10.

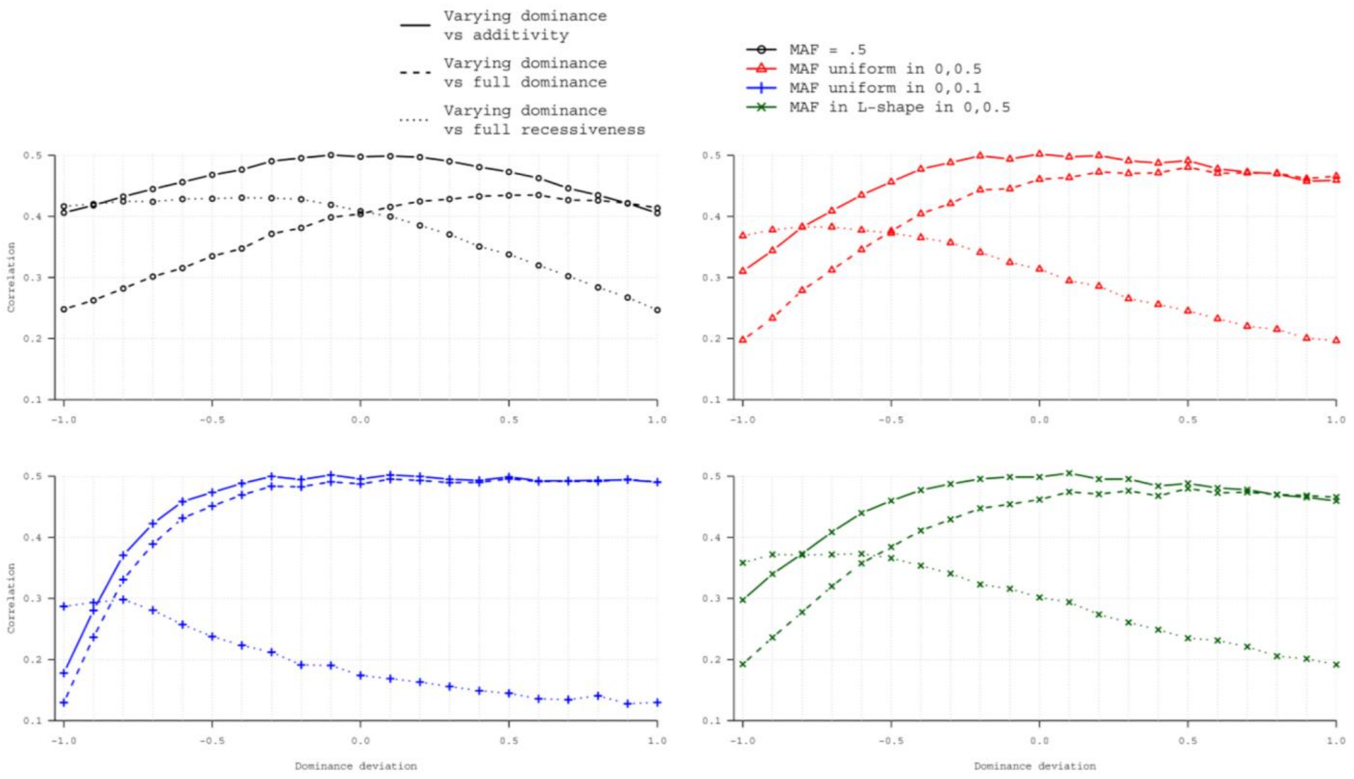

Supplementary Figure 10. Simulated correlation (*y-axis*) due to genetics with different MAF, and where one phenotype's genotypic effect varies in dominance deviation (the *h* as proportion of *d*; *x-axis*) while the other's is fixed as purely additive or with maximal dominance or recessive deviation. Negative values indicate that the minor allele is recessive, positive that it is dominant.

We observe allelic frequency affect in all simulated correlations except where both individual's genotypic expression is completely additive (where the correlation between individuals is 0.5 for all MAF distributions). Further, we can see that scenario A has a higher correlation than scenario B and C, except for when the dominance deviation goes towards its extremes, greater/lesser than approximately ±0.8. This is most clearly seen for Scenario B, where the minor allele is recessive.

In reality, we would not have any way of knowing the MAF distributions for contributing loci, nor would we know the dominance deviations (which would vary between loci).

Suggested intuitive solution

We suggest that estimation of genetic correlation based on twin data based on an intuitive solution. We have observed that correlating a phenotype with varying dominance deviation between twins will produce a resulting correlation which lies between pure additivity (i.e., correlation = 0.5) and maximal dominance deviation for either the major or minor allele (varying resulting correlation depending on MAF) – except for when the dominance deviations are nearing its maxima or minima. Hence, if the classic twin model correctly captures the additive genetic effects (A) and dominance deviations (D) for each trait separately, we can suggest an intuitive modeling approach to estimate the genetic correlation between the two which relies on placing the resulting correlation in between that of A and D alone.

Expected genetic correlation

To be able to assess the performance of an estimating procedure of the genetic correlation we need to know the genetic correlation if both phenotypes were expressed in one individual. Since the solution when allele frequencies are unequal quickly become unruly, we simulate this is a similar fashion as in *Section 2* above, using the same 'L'-shaped MAF. We simulated using 100,000 individuals per each combination of values from −1 to 1 by steps of 0.1 – in Supplementary Figure 11 the result is presented.

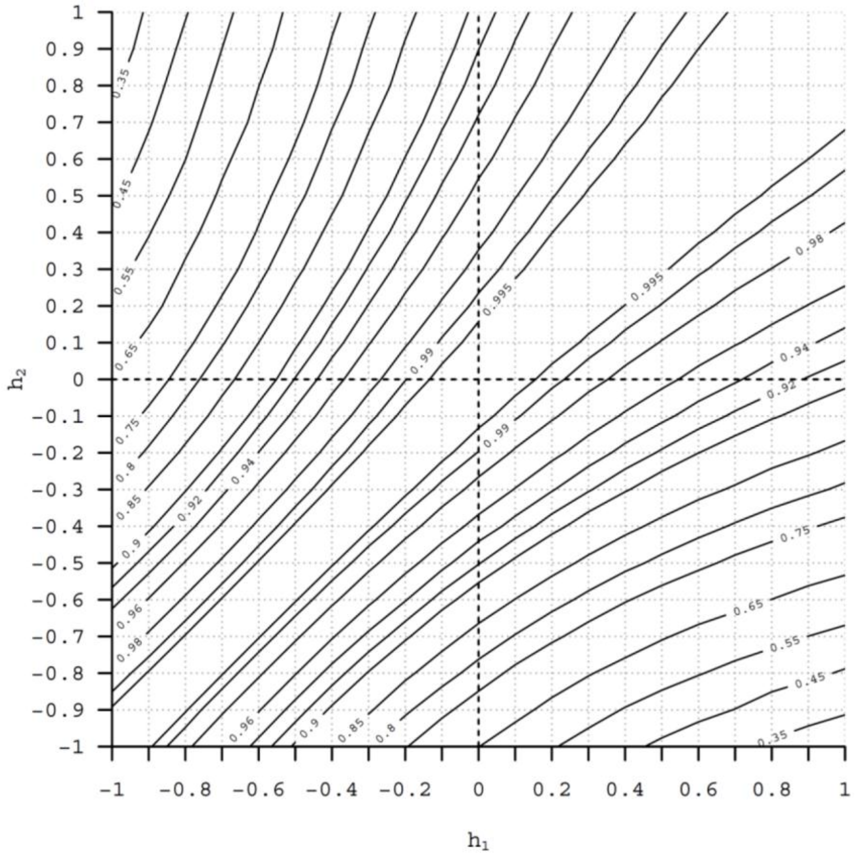

Supplementary Figure 11. Simulation for estimating genetic correlation. The contour plot shows resulting genetic correlation under different amounts of dominance deviation (positive) and recessive deviations (negative) for the minor allele.

We can observe that produced correlations deviate from 1 as the dominance deviations (or recessive ditto) moves away from 0 differently in the two phenotype's genotypic effects. The genetic correlation becomes lowest when one phenotype has more dominance deviation for the minor allele and the other has more recessive deviations. We can also see that it matters which of the major or minor allele is more dominant, where the minor allele being dominant is less problematic.

Suggested estimating procedure

Per the classic twin model – with *A*, without *D* and *C* (the 'shared environment' contribution), but with *E*, the individually unique contributions to variance not shared between individuals (often referred to as an *AE*-model) – we may model the covariance between opposite sexed DZ twins (osDZ) as (see e.g. Neale and Maes [1]; here sub-index ending with *f* indicated the female twin and sub-index ending with *m* indicated the male twin):

$$Cov\left(\begin{bmatrix} Y_1 \\ Y_2 \end{bmatrix}\right)_{osDZ} = \begin{bmatrix} \sigma_{Af}^2 + \sigma_{Ef}^2 & r_{fm} 0.5 \sigma_{Af} \sigma_{Am} \\ r_{fm} 0.5 \sigma_{Af} \sigma_{Am} & \sigma_{Am}^2 + \sigma_{Em}^2 \end{bmatrix} \quad (7)$$

The model fitting would estimate the r_{fm} as the genetic correlation between males and females, appropriately bounded between −1 and 1. A direct translation into situation where the model is an *ADE* model is

$$Cov\left(\begin{bmatrix} Y_1 \\ Y_2 \end{bmatrix}\right)_{osDZ} = \begin{bmatrix} \sigma_{Af}^2 + \sigma_{Df}^2 + \sigma_{Ef}^2 & r_{fm} 0.5 \sigma_{Af} \sigma_{Am} + 0.25 \sigma_{Df} \sigma_{Dm} \\ r_{fm} 0.5 \sigma_{Af} \sigma_{Am} + 0.25 \sigma_{Df} \sigma_{Dm} & \sigma_{Am}^2 + \sigma_{Dm}^2 + \sigma_{Em}^2 \end{bmatrix} \quad (8)$$

However, in this setup the estimated genetic correlation is not allowing *D* to contribute to estimation of r_{fm}. A natural expansion with the inclusion of *D* is

$$Cov\left(\begin{bmatrix} Y_1 \\ Y_2 \end{bmatrix}\right)_{osDZ} = \begin{bmatrix} \sigma_{Af}^2 + \sigma_{Df}^2 + \sigma_{Ef}^2 & r_{fm}\left(0.5\sigma_{Af}\sigma_{Am} + 0.25\sigma_{Df}\sigma_{Dm}\right) \\ r_{fm}\left(0.5\sigma_{Af}\sigma_{Am} + 0.25\sigma_{Df}\sigma_{Dm}\right) & \sigma_{Am}^2 + \sigma_{Dm}^2 + \sigma_{Em}^2 \end{bmatrix} \quad (9)$$

The assumption implicitly encoded here is that the same subset of loci having dominance deviations in one sex's genotypic expression also has dominance deviation expression in the other sex, while additive effects in one sex does not correlate with dominance deviations in the other sex. As shown above, if we deviate from this assumption, we will have different resulting correlations due to genetic effects. As an extreme example, from a twin modelling view-point, suppose the female trait has an estimated 0 *A* contribution according to the model and some *D* contribution, while the male has only *A* contribution and no *D*; the result would be that there's no way the model could ascribe any correlation between sexes to genetics (and the modelled r_{fm} would take on any value between -1 and 1 with equal likelihood). This is obviously not appropriate, since the same locus could contribute in a pure additive way for one sex's phenotype and a dominant way for the other sex's phenotype. We therefore suggest that the correlation can be modelled as (note, same equation as in *Introduction*)

$$Cov\left(\begin{bmatrix} Y_1 \\ Y_2 \end{bmatrix}\right)_{osDZ} = \begin{bmatrix} \sigma_{Af}^2 + \sigma_{Df}^2 + \sigma_{Ef}^2 & r_{fm}\left(0.5\sigma_{Af}\sigma_{Am} + 0.25\sigma_{Df}\sigma_{Dm} + 0.25\sigma_{Af}\sigma_{Dm} + 0.25\sigma_{Df}\sigma_{Am}\right) \\ r_{fm}\left(0.5\sigma_{Af}\sigma_{Am} + 0.25\sigma_{Df}\sigma_{Dm} + 0.25\sigma_{Af}\sigma_{Dm} + 0.25\sigma_{Df}\sigma_{Am}\right) & \sigma_{Am}^2 + \sigma_{Dm}^2 + \sigma_{Em}^2 \end{bmatrix} \quad (1)$$

This suggestion is based on the observed simulated correlations behavior towards the extremes of the dominance deviation (Supplementary Figure 10), where correlation due to genetics between DZ twins seem to be most affected by differences in dominance deviations. Not allowing genetics to contribute to the correlation across individuals *A and D sources*, may cause bias; and, towards the extremes, the correlation between additive and dominant/recessive genetic effects resemble that of dominance-to-dominance correlation behavior (although, not an exact correspondence between the two). As such, we expect the performance of the estimating procedure to be most suitable for scenarios where the *A* and *D* contributions to phenotypic variance differ considerable between males and females.

To investigate the performance of this suggested modelling approach we performed a series of simulations. For each simulation we used the above approach of simulating parental alleles and drawing offspring alleles, and then we created same sexed MZ and DZ twin pairs in addition to the opposite sexed DZ pairs. We assumed 7000 pairs for each sex-zygosity combination. Additionally, we estimated the resulting genetic correlation, to be able to compare the produced correlation with. We added an individually unique variation (the *E*), drawn from a random normal distribution with the same variance as the simulated genetic variance separately by sex, and used the classic twin methodology with sex-limitation models to the data. We fitted the models using equations (1) (our suggestion), (9), (8), and (7), to provide an overall picture of the performance of different approaches. We a. investigated the full range of dominance deviations (all combinations of the *h*'s between −1 and 1 by 0.25 steps), b. investigated the situation where one phenotype is additive and the other has dominance deviations (the h_1 in −1.0 to −0.8 and 0.8 to 1.0 in 0.05 steps, the h_2 fixed at 0), and c. did a more thorough investigation on the extreme values (the h_1 in −1.0 to −0.8 and h_2 in 1.0 to 0.8 in 0.05 steps) – for each combination the simulation was run 10 times. In the simulation we used the same 'L'-shaped MAF as above. In Supplementary Figures 12a, 12b, and 12c the results of these three sets of simulations are shown. We plot the difference between estimated r_{fm} and calculated true r_G as an estimate of bias in the separate estimating approaches.

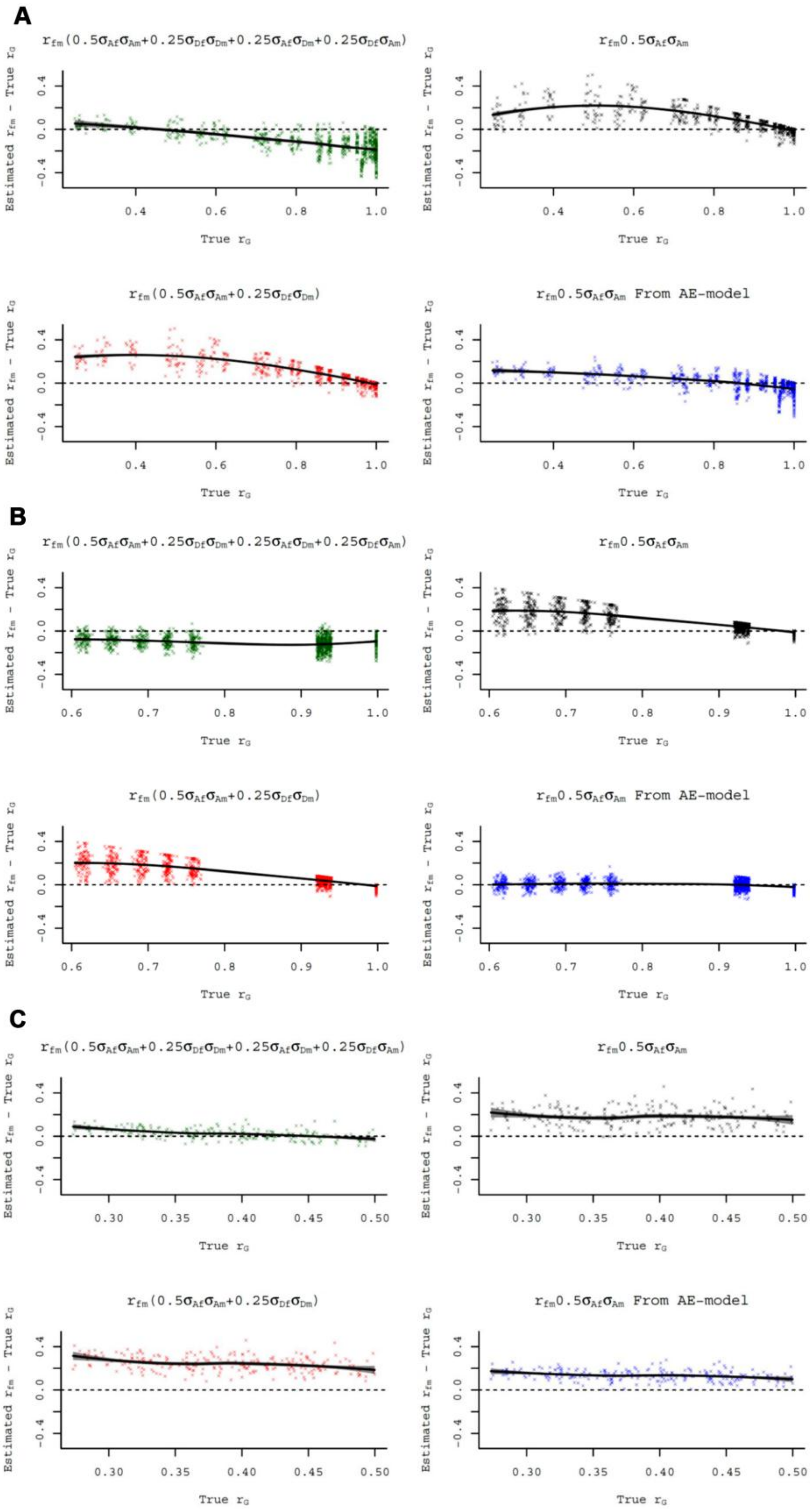

Supplementary Figure 12. Performance of different estimation procedures in a simulation. A locally smoothed polynomial regression line is fitted to each scenario (using 'loess' function in R). (**A**) Full range of dominance deviations. (**B**) Dominance deviations versus additive. (**C**) Dominance deviations towards extreme.

We summarize the behavior of the four different estimating approaches in Supplementary Table 12. We observe the following features of the four different estimating approaches:

1. Neither estimating approach has as good performance throughout the dominance deviation range.

2. Our suggested estimating approach has best performance in the most extreme scenarios.

3. Our suggested estimating approach has a negative bias for most scenarios, and only slightly positive bias when dominance deviations are towards the very extremes.

4. All standard estimating approach (i.e., the three simulated estimating approach except our suggestion) generally has a upwards bias, towards 1.0.

5. The modelling approach of the AE-model perform better than other estimating approach, except for the very extremes, with regard to bias (low mean squared error) but precision may be overestimated, i.e. too low standard errors (with relatively lower coverage probability compared with standard approaches).

Supplementary Table 12. Performance over different scenarios for the investigated estimating procedures.

	**a. Full range of dominance deviations**	**b. Dominance deviations versus additive**	**c. Dominance deviations towards extreme**
Mean squared error			
$r_{fm}\left(0.5\sigma_{Af}\sigma_{Am}+0.25\sigma_{Df}\sigma_{Dm}+0.25\sigma_{Af}\sigma_{Dm}+0.25\sigma_{Df}\sigma_{Am}\right)$	0.029	0.015	0.003
$r_{fm}0.5\sigma_{Af}\sigma_{Am}+0.25\sigma_{Df}\sigma_{Dm}$	0.016	0.020	0.038
$r_{fm}\left(0.5\sigma_{Af}\sigma_{Am}+0.25\sigma_{Df}\sigma_{Dm}\right)$	0.020	0.021	0.063
$r_{fm}0.5\sigma_{Af}\sigma_{Am}$ from AE-model	0.007	0.002	0.021
Mean error			
$r_{fm}\left(0.5\sigma_{Af}\sigma_{Am}+0.25\sigma_{Df}\sigma_{Dm}+0.25\sigma_{Af}\sigma_{Dm}+0.25\sigma_{Df}\sigma_{Am}\right)$	-0.126	-0.103	0.024
$r_{fm}0.5\sigma_{Af}\sigma_{Am}+0.25\sigma_{Df}\sigma_{Dm}$	0.074	0.100	0.178
$r_{fm}\left(0.5\sigma_{Af}\sigma_{Am}+0.25\sigma_{Df}\sigma_{Dm}\right)$	0.086	0.105	0.242
$r_{fm}0.5\sigma_{Af}\sigma_{Am}$ from AE-model	-0.001	0.001	0.134
Proportion negative bias			
$r_{fm}\left(0.5\sigma_{Af}\sigma_{Am}+0.25\sigma_{Df}\sigma_{Dm}+0.25\sigma_{Af}\sigma_{Dm}+0.25\sigma_{Df}\sigma_{Am}\right)$	0.872	0.935	0.318
$r_{fm}0.5\sigma_{Af}\sigma_{Am}+0.25\sigma_{Df}\sigma_{Dm}$	0.234	0.112	0.008
$r_{fm}\left(0.5\sigma_{Af}\sigma_{Am}+0.25\sigma_{Df}\sigma_{Dm}\right)$	0.229	0.096	0.000
$r_{fm}0.5\sigma_{Af}\sigma_{Am}$ from AE-model	0.499	0.463	0.000
Coverage probability			
$r_{fm}\left(0.5\sigma_{Af}\sigma_{Am}+0.25\sigma_{Df}\sigma_{Dm}+0.25\sigma_{Af}\sigma_{Dm}+0.25\sigma_{Df}\sigma_{Am}\right)$	0.602	0.721	0.895
$r_{fm}0.5\sigma_{Af}\sigma_{Am}+0.25\sigma_{Df}\sigma_{Dm}$	0.862	0.895	0.448
$r_{fm}\left(0.5\sigma_{Af}\sigma_{Am}+0.25\sigma_{Df}\sigma_{Dm}\right)$	0.755	0.841	0.054
$r_{fm}0.5\sigma_{Af}\sigma_{Am}$ from AE-model	0.768	0.965	0.192

CONCLUSIONS

In a scenario with diverging dominance and additive contributions to phenotypic variance in males and females using the classic twin model, the standard estimating approaches has the feature that they overestimate the genetic correlation between males and females (which we here call r_{fm}). Thus, even if there is a genetic correlation lower than 1.0 the estimating approaches will have a lower likelihood of detecting it since they are biased upwards. In contrast, using our suggested approach will produce an estimate which, if it is biased at all, will be biased downwards. Therefore, if a model fitted with our estimating approach produces a r_{fm} which is not statistically significant, it is likely a true null finding. Additionally, if fitting an *AE*-model is not appropriate (e.g., due to poor model fit), our suggested estimating approach has a lower mean squared error than standard estimating approach in extreme scenarios (with a larger proportion of negative bias).

REFERENCE

1. Neale M, Maes HH. Methodology for genetic studies of twins and families. Secondary Methodology for genetic studies of twins and families. http://ibgwww.colorado.edu/workshop2006/cdrom/HTML/book2004a.pdf
